# Prediction Model for Flake Line Defects in Metallic Injection Molding: Considering Skin-Core Velocity and Alignment

**DOI:** 10.3390/polym17020245

**Published:** 2025-01-20

**Authors:** Seungkwon Choi, Donghwi Park, Seungcheol Lee, Minho Song, Naksoo Kim

**Affiliations:** 1 Department of Mechanical Engineering, Sogang University, Seoul 04107, Republic of Korea; csg7669@sogang.ac.kr (S.C.); pdhwi93@u.sogang.ac.kr (D.P.); lsc4082@sogang.ac.kr (S.L.); 2 Manufacturing Solution Division, Hyundai Motor Company, Uiwang-si 16082, Republic of Korea; mino@kia.com

**Keywords:** metallic injection molding, flake line defects, velocity model, misalignment index, aluminum flakes, skin-core interaction, appearance defect

## Abstract

Metallic injection molding combines aluminum flake metallic pigments with polymers to directly produce components with metallic luster, improving production efficiency and reducing environmental impact. However, flake line defects that occur in regions where ribs or flow paths intersect remain a significant challenge. This study proposes a velocity model that considers the flow characteristics between the surface and core layers and an alignment model that incorporates the orientation of aluminum flakes to predict appearance defects. Through this approach, the mechanisms of appearance defect formation were systematized, and the appearance defects caused by flow velocity differences between the surface and core layers, flake alignment uniformity, and reflection angles were visualized. Both prediction models demonstrated a 50% prediction accuracy, successfully identifying two out of four observed defects. This research addresses the limitations of previous prediction methods, which only considered the surface layer, by introducing a novel approach that accounts for the core layer. It is expected to contribute to reducing defects and improving quality in industries requiring high-quality metallic appearances.

## 1. Introduction

Metallic injection molding is a technology that blends metallic pigments, such as aluminum flakes, with polymers to directly mold parts with metallic luster and reflective effects. This process eliminates the need for post-treatments such as painting or plating, simplifying the manufacturing process while reducing production costs and environmental impact. For these reasons, it is widely used in industries where appearance quality is critical, such as home appliances, automotive interiors, and mobile device cases [1,2].

Despite its advantages, appearance defects, particularly flake line defects, remain an unresolved issue in metallic injection molding. These defects primarily arise from the uneven alignment of metallic flakes on the surface layer. During the molding process, when molten polymers flow along the mold walls, flakes align parallel to the surface, resulting in a bright and uniform gloss. However, in regions where flows recombine after passing obstacles such as ribs or holes, the complex flow patterns disrupt the uniform alignment of flakes, causing them to exhibit irregular or tilted orientations. These irregularities arise from sudden changes in velocity gradients and shear stress during the recombination process. As a result, the reflective properties are significantly degraded, and the perceived color intensity is reduced, creating visible defects such as streaks or an uneven gloss [2,3,4,5,6].

Previous studies have primarily focused on predicting appearance defects based on flake orientation in the surface layer. Toshiki Sasayama predicted appearance defects by calculating the orientation of flake pigments exclusively in the surface layer [3,7]. Similarly, Se Lyn Kim utilized the Folgar-Tucker model to calculate the orientation of aluminum flakes and predict appearance defects [8].

However, in practice, the flow differences between the core and surface layers play a significant role in the formation of appearance defects. In the core layer, low shear rates and complex flow recombination result in irregular alignment, while the surface layer achieves predominantly parallel alignment due to a high shear flow near the mold wall. These differences in flow characteristics lead to uneven flake alignment, manifesting as appearance defects such as reduced gloss and diminished color intensity [9,10].

This study develops a model to predict flake line defects caused by velocity changes during the filling process, considering flow characteristics in both the surface and core layers. Additionally, the prediction model improves accuracy by incorporating the angle between the surface and the flakes into traditional flake orientation-based prediction methods. To evaluate and validate the prediction model, the predicted defect locations were compared with the actual defect locations observed under a microscope. This process not only assessed the model’s accuracy but also provided insights into the mechanisms behind defect formation, allowing for a deeper understanding of the relationship between flow characteristics and flake alignment. These findings aim to enhance the reliability and applicability of the model in practical industrial settings.

## 2. Numerical Method

### 2.1. Velocity Model

**Figure 1:**
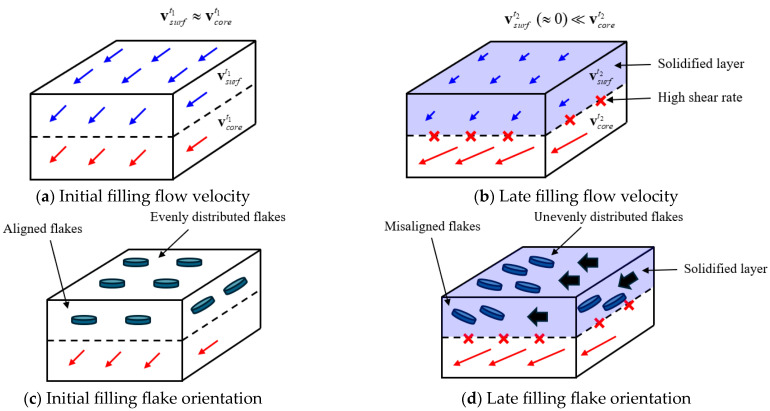
Shear phenomenon between surface and core flow due to changes in flow velocity during the charging process: (**a**) initial filling flow velocity, (**b**) late filling flow velocity, (**c**) initial filling flake orientation, (**d**) late filling flake orientation.

During the filling process of injection molding, the flow velocity of the surface layer $v_{surf}$ is faster than the core layer velocity $v_{core}$ due to viscous flow. At the beginning of the filling process, the difference between the surface-layer and core-layer velocities is negligible, as shown in Figure 1a, so the flakes are aligned in the same direction and distributed uniformly, as shown in Figure 1c. However, as time passes, a thin surface solidification layer forms on the surface layer of the product, as shown in Figure 1b, and a difference in the magnitude and direction of velocity between the surface-layer and the core-layer flows occurs. This change in flow velocity affects the surface solidification layer, and shear stress acts in this process, causing shear deformation between the flows due to the change in velocity.

As shown in Figure 1d, the orientation and density of the surface-layer flakes change in the region where the shear deformation occurs, and they are unevenly distributed and aligned in different directions. This results in visible defects.

In this study, two defect condition functions are defined to simulate the flake line caused by this shear phenomenon. The first is the defect condition function $f_{v}$ for the average velocity change. It is a function of the average velocity change Δv of the initial surface-flow velocity $v_{surf}$ and the late core-layer velocity $v_{core}$ for a node during the charging time, and is calculated by Equations (1) to (3).

However, $v_{core}$ refers to the vector projected onto the surface of the core-layer flow vector and ignores the velocity change in the direction of the product thickness. ${\left\|\Delta v\right\|}_{\mathrm{mean}}$ is the average value of the average velocity change for all nodes, and $\sigma_{v}$ is the standard deviation value of the average velocity change. A higher $f_{v}$ value indicates a more pronounced velocity difference between the surface flow and core flow, increasing the likelihood of appearance defects.

(1) $$\Delta v_{i}={\left(v_{core}-v_{surf}\right)}_{mean}\;$$

(2) $${\left\|\Delta v\right\|}_{\mathrm{mean}}=\frac{1}{N}\sum_{i=1}^{N}\left\|\Delta v_{i}\right\|\;\;\;,\;\;\;\sigma_{v}=\sqrt{\frac{1}{N}\sum_{i=1}^{N}{\left(\left\|\Delta v_{i}\right\|-{\left\|\Delta v\right\|}_{\mathrm{mean}}\right)}^{2}}$$

(3) $$f_{v}=\frac{\left\|\Delta v_{i}\right\|}{\sigma_{v}}$$

The second is the defect condition function $f_{\theta }$ for the difference in the flow direction of the average velocity change. This function calculates the angle θ between the average velocity change vectors of adjacent nodes *i* and *j*, as defined by Equations (4) to (6).

$\theta_{\mathrm{mean}}$ is the average value of the angle between the average velocity change vectors for all nodes, and $\sigma_{\theta }$ is the standard deviation of the angle between the average velocity change vectors. A higher $f_{\theta }$ value signifies a more abrupt difference in flow direction between adjacent flows, increasing the likelihood of appearance defects.

(4) $$\theta =\cos^{-1}\left(\frac{\Delta v_{i}\cdot \Delta v_{j}}{\left\|\Delta v_{i}\right\|\cdot \left\|\Delta v_{j}\right\|}\right)\;$$

(5) $$\theta_{\mathrm{mean}}=\frac{1}{N}\sum_{k=1}^{N}\theta_{k}\;,\;\sigma_{\theta }=\sqrt{\frac{1}{N}\sum_{k=1}^{N}{\left(\theta_{k}-\theta_{\mathrm{mean}}\right)}^{2}}$$

(6) $$f_{\theta }=\frac{\theta_{k}}{\sigma_{\theta }}$$

Finally, to define the defect evaluation function $F_{defect\_velocity}$ that determines the occurrence of an appearance defect, two defect conditions, $F_{v}$ and $F_{\theta }$, were each normalized as in Equation (7) and a defect evaluation function, Equation (8), was created by adding weights α and β to each normalized defect condition.

(7) $$F_{v}=\frac{f_{v}-f_{v,\;\min}}{f_{v,\;\max}-f_{v,\;\min}},\;\;\;F_{\theta }=\frac{f_{\theta }-f_{\theta ,\;\min}}{f_{\theta ,\;\max}-f_{\theta ,\;\min}}$$

(8) $$F_{defect\_velocity}=\alpha \times F_{v}+\beta \times F_{\theta }$$

**Figure 2:**
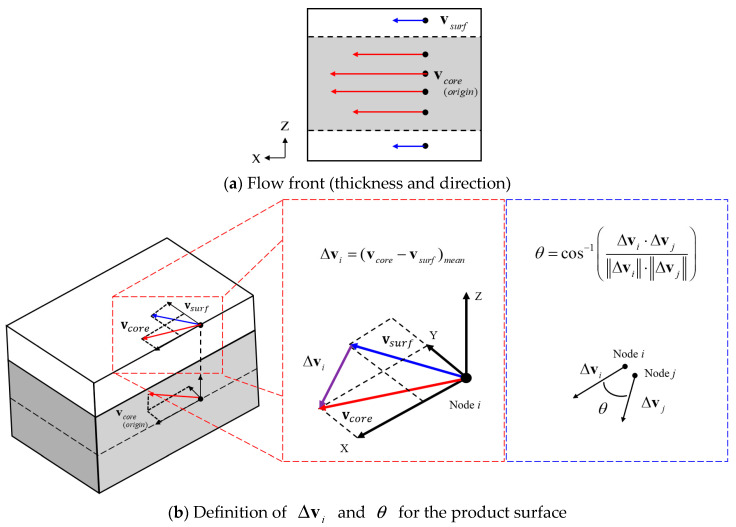
The definition of $\Delta v_{i}$ and θ due to the change in velocity between the surface and core flow. (**a**) Flow front (thickness and direction), and (**b**) the definition of $\Delta v_{i}$ and θ for the product surface.

Figure 2 shows the definition of the average velocity change Δv caused by the velocity difference between the surface and core flows, and the angle θ between the average velocity change vector and the surface.

To account for the unique shape and flow characteristics of the product, the defect evaluation function has been added to the conditions of the region where it is not applied exceptionally, so that only the actual appearance defects can be more accurately predicted. The following are the main conditions added:

Condition 1: In curved regions such as filets or at the edges of a product, abrupt velocity changes can lead to directional changes in flow vectors. However, these changes are stabilized through the mutual cancelation of vectors, maintaining the overall stability of the flow. This stable flow environment ensures that metallic flakes are uniformly aligned, preventing appearance defects [3,11,12,13,14,15]. Equation (9) presents the formula for Condition 1. In this study, the mutual cancelation effect is defined as occurring when it exceeds the top 10% threshold of the condition.

(9) $$\Delta v>\;0.9\times \Delta v_{\max}\;,\;\theta >0.9\times \theta_{\;\max}\;$$

Condition 2: If the change in velocity vectors is minimal in the planar region, the condition is classified as normal, indicating a region where no defects are expected to occur. When the angle between the average velocity change vectors is low, the flake arrangement remains stable, minimizing the occurrence of appearance defects [16,17]. Equation (10) presents the mathematical expression for Condition 2. In this study, the condition is set to be insignificant if it is less than the lower 10%.

(10) $$\theta <0.1\times \theta_{\;\max}\;$$

Condition 3: Regions with small average velocity change differences are classified as defect-free zones. A uniform velocity distribution plays an important role in maintaining a consistent surface appearance by preventing sudden changes in the flake arrangement [18,19,20,21,22]. Equation (11) shows the expression for Condition 3. In this study, regions are classified as having small average velocity change differences if the value falls below the lower 10%.

(11) $$\Delta v_{i}-\Delta v_{j}<0.1\times {\left(\Delta v_{i}-\Delta v_{j}\right)}_{\;\max}\;,\;\;\;\theta_{i}-\theta_{j}<0.1\times {\left(\theta_{i}-\theta_{j}\right)}_{\;\max}\;$$

In the velocity model of this study, the accuracy of defect prediction is measured by excluding regions that meet three conditions from the defect evaluation function.

### 2.2. Alignment Model

The alignment uniformity of flakes is a critical factor that directly affects the reflective properties of the product surface. Higher alignment uniformity results in consistent reflectivity and a bright gloss, while lower alignment uniformity leads to irregular flake orientations, reducing reflectivity and creating an uneven gloss. At boundaries where alignment uniformity varies, differences in reflectivity arise, causing appearance defects such as flake lines [23,24,25].

In this study, the misalignment of flakes on the surface was quantified as the Misalignment Index (*M.I*.), as shown in Equation (12). Orientation tensor values for each node were extracted from injection molding simulations, and these tensors were projected onto the surface’s infinitesimal area to isolate surface alignment components. Subsequently, the eigenvalues of the projected matrix were calculated to determine the *M.I.*

**Figure 3:**
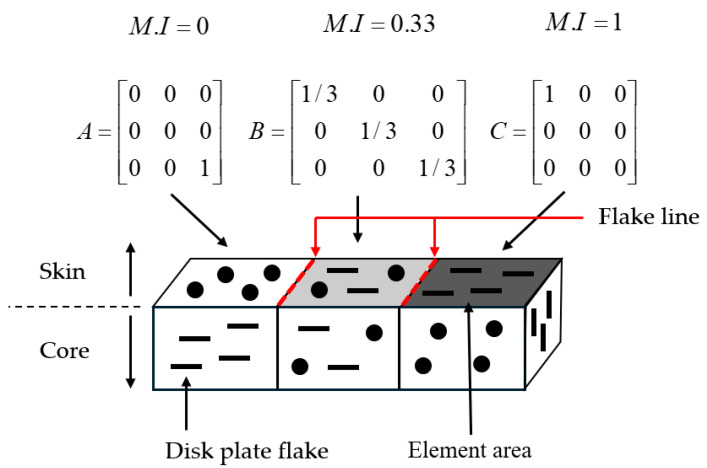
Flake orientation alignment degree-dependent color differences and *M.I.* (Misalignment Index) values.

Figure 3 illustrates the mechanism of color differences caused by flake alignment uniformity and presents the *M.I.* values corresponding to each tensor configuration in different colors. Higher *M.I*. values correspond to lower reflectivity, resulting in darker colors and a higher likelihood of defects. Notably, at boundaries where *M.I*. values between adjacent regions exhibit abrupt changes, there is an increased likelihood of pronounced appearance defects.

In this process, the analysis was conducted using the Moldflow program (Moldflow 2019) to calculate the orientation tensor value of each node, and the Folgar-Tucker model was used. The Folgar-Tucker model is widely used as a numerical approach to predict fiber orientation during injection molding. In Equation (13), $\dot{\gamma }$ is the strain rate tensor, a is the fiber orientation tensor, $C_{l}$ is the interaction coefficient, and λ is the orientation factor. This model was developed based on Jeffery’s equation, adding isotropic rotational diffusion to account for the interaction between fibers.

The model calculates how the fibers move and deform under hydrodynamic flow conditions, including w and $\dot{\gamma }$. Interactions between fibers and the effect of fiber geometry on orientation are effectively modeled by $C_{l}$ and λ [26,27,28].

(12) $$M.I=\sqrt{\frac{1}{2}\left({\left(\lambda_{1}-\lambda_{2}\right)}^{2}+{\left(\lambda_{2}-\lambda_{3}\right)}^{2}+{\left(\lambda_{3}-\lambda_{1}\right)}^{2}\right)}$$

(13) $$\frac{Da_{ij}}{Dt}=-\frac{1}{2}\left(w_{ik}a_{kj}-a_{ik}w_{kj}\right)+\frac{1}{2}\lambda \left({\dot{\gamma }}_{ik}a_{kj}+a_{ik}{\dot{\gamma }}_{kj}-2a_{ijkl}\right)+2C_{l}\dot{\gamma }\left(\delta_{ij}-3a_{ij}\right)$$

In addition, the difference in the angle between the flake and the surface of the product causes a difference in the intensity of light reflection, and defects occur in regions where the reflectivity decreases. The greater the angle between the normal vector $\vec{n_{f}}$ of the flake and the normal vector $\vec{n_{s}}$ of the surface, the less light is reflected, resulting in a darker color. This phenomenon is especially noticeable in complex shapes such as ribs and holes, and defects occur at the interface where color differences occur [5].

**Figure 4:**
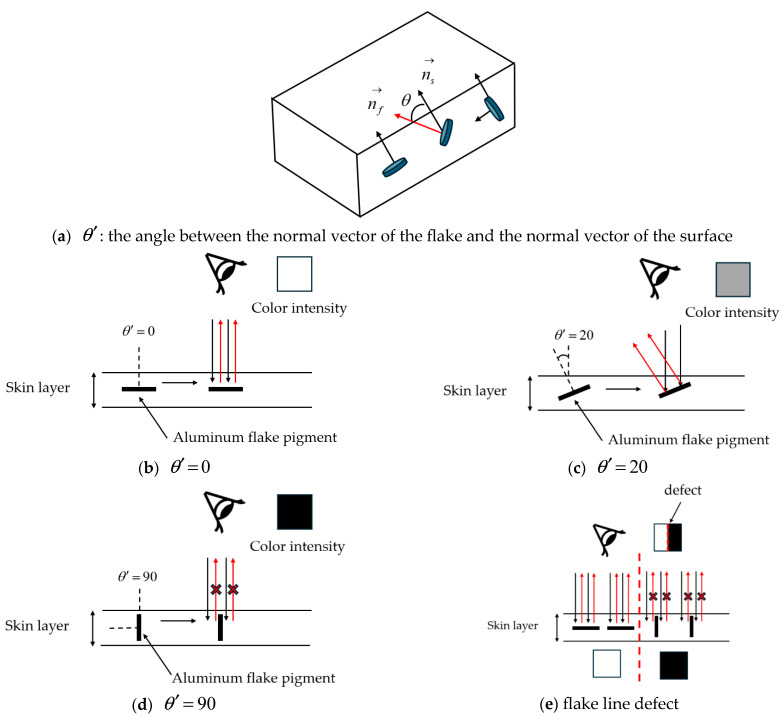
The color difference mechanism is based on the difference in the angle between the flake and the surface. (**a**) $\theta^{\prime }$: the angle between the normal vector of the flake and the normal vector of the surface; (**b**) $\theta^{\prime }=0$; (**c**) $\theta^{\prime }=20$; (**d**) $\theta^{\prime }=90$; and (**e**) flake line defect.

In this study, the angle difference between the flake and the surface was quantified as $\theta^{\prime }$, as shown in Equation (14). The smaller the $\theta^{\prime }$, the more parallel the flake and the surface, and the higher the reflectance. Conversely, the greater the $\theta^{\prime }$, the more perpendicular the flake and the surface, and the lower the reflectance. This mechanism shows the color difference according to the angle between the flake and the surface, as shown in Figure 4.

Higher $\theta^{\prime }$ values correspond to lower reflectivity, resulting in darker colors and an increased likelihood of defects. In particular, at boundary regions where there is a sudden difference in $\theta^{\prime }$ values between areas with relatively uniform $\theta^{\prime }$, the likelihood of pronounced appearance defects significantly increases. Additionally, The $\theta^{\prime }$ and the density of flakes interact and influence the optical properties of the product. The more parallel the flakes are to the surface, the greater the reflectivity of light, and a higher density increases the number of flakes contributing to reflection, further enhancing this effect.

(14) $$\theta^{\prime }=\cos^{-1}\left(\frac{n_{f}\cdot n_{s}}{\left\|n_{f}\right\|\cdot \left\|n_{s}\right\|}\right)$$

In this study, two defect condition functions were defined to simulate the flake line based on the color difference caused by the flake alignment and the angle difference with the surface. The first is the defect condition function $f_{M.I}$ for the non-alignment index *M.I.* and is calculated by Equations (15) and (16). $M.I_{\mathrm{mean}}$ is the average value of *M.I*. for all nodes and $\sigma_{M.I}$ is the standard deviation value of *M.I*. The higher the value of $f_{M.I}$, the lower the alignment and the darker the color.

(15) $$M.I_{\mathrm{mean}}=\frac{1}{N}\sum_{i=1}^{N}M.I_{i}\;,\;\sigma_{M.I}=\sqrt{\frac{1}{N}\sum_{i=1}^{N}{\left(M.I_{i}-M.I_{\mathrm{mean}}\right)}^{2}}$$

(16) $$f_{M.I}=\frac{M.I_{i}}{\sigma_{A.I}}$$

The second is the defect condition function $f_{angle}$ for the difference in the angle between the flake and the surface, which is calculated by Equations (17) and (18). ${\theta^{\prime }}_{\mathrm{mean}}$ is the average value of $\theta^{\prime }$ for all nodes, and $\sigma_{angle}$ is the standard deviation value of $\theta^{\prime }$. The higher the $f_{angle}$ value, the greater the angle difference between the flake and the surface, which means that the color appears darker.

(17) $${\theta^{\prime }}_{\mathrm{mean}}=\frac{1}{N}\sum_{i=1}^{N}{\theta^{\prime }}_{i}\;,\;\sigma_{angle}=\sqrt{\frac{1}{N}\sum_{i=1}^{N}{\left({\theta^{\prime }}_{i}-{\theta^{\prime }}_{\mathrm{mean}}\right)}^{2}}$$

(18) $$f_{angle}=\frac{{\theta^{\prime }}_{i}}{\sigma_{angle}}$$

Finally, to define the defect evaluation function $F_{defect\_velocity}$ that determines the occurrence of an appearance defect, the two defect conditions $F_{M.I}$ and $F_{angle}$ were normalized as shown in Equation (19), and the defect evaluation function Equation (20) was created by adding weights γ and κ to each normalized defect condition.

(19) $$F_{M.I}=\frac{f_{M.I}-f_{M.I,\;\min}}{f_{M.I,\;\max}-f_{M.I,\;\min}},\;\;\;F_{angle}=\frac{f_{angle}-f_{angle,\;\min}}{f_{angle,\;\max}-f_{angle,\;\min}}$$

(20) $$F_{defect\_alignment}=\gamma \times F_{M.I}+\kappa \times F_{angle}$$

Due to the unique shape of the product, the defect evaluation function was modified to include conditions for regions where it is exceptionally not applied, enabling the more accurate prediction of actual appearance defects. The following are the main conditions that have been added:

Condition 1: On curved surfaces such as filet regions, the flake alignment is irregular due to the curvature and complex flow, and the angle between the flake and the surface is not constant, resulting in excessive misalignment and a large angle. However, on curved surfaces, there are various reflectance and irregular characteristics depending on the angle, and the alignment or angle is not constant in a specific region. Therefore, there is no sudden difference in alignment or angle between regions. Due to this curvature property, there are no visible defects in appearance due to distinct color differences [23,24,25,26]. Equation (21) shows the equation for Condition 1. In this study, we set the condition to show the characteristics of non-alignment and excessive irregularity of angles when the condition exceeds the top 10%.

(21) $$M.I>\;0.9\times M.I_{\max}\;,\;\theta^{\prime }>0.9\times {\theta^{\prime }}_{\;\max}\;$$

Condition 2: In regions where non-alignment or a sharp difference in the angle between flakes and the surface does not occur, color differences appear gradually, so there is no visual appearance defect due to a clearly visible color difference [23,24,25,26,29,30,31,32]. Equation (22) shows the expression for Condition 2. In this study, we consider a region where color differences appear gradually if each condition is less than the bottom 10%.

(22) $$M.I_{i}-M.I_{j}<0.1\times {\left(M.I_{i}-M.I_{j}\right)}_{\;\max}\;,\;\;\;{\theta^{\prime }}_{i}-{\theta^{\prime }}_{j}<0.1\times {\left({\theta^{\prime }}_{i}-{\theta^{\prime }}_{j}\right)}_{\;\max}\;$$

The alignment model enhances defect prediction accuracy by excluding regions with a low likelihood of defect occurrence. Curved surfaces with excessively irregular misalignment and angles (Condition 1) and regions where color differences are not distinctly visible (Condition 2) are excluded from the defect evaluation function, allowing the model to focus on areas where noticeable defects occur, thereby improving prediction precision.

## 3. Experiment

**Figure 5:**
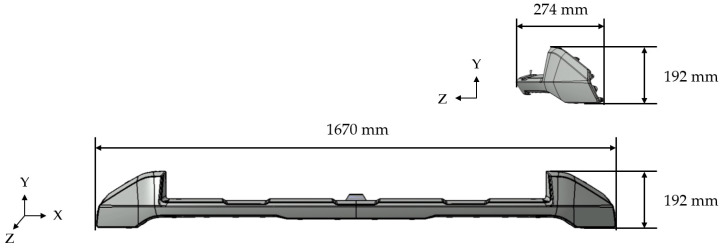
Specimen geometry.

The injection of the actual product was carried out to compare the results with the predicted appearance defects. The material used was Daewon Chemical’s JR3512HM (TPO) with 3.3 wt% aluminum flake (average diameter 60 μm, thickness 0.6 μm) added in the form of a circular plate. The product used was Kia’s Carnival front skid plate, and its shape is shown in Figure 5. The injection conditions were a mold temperature of 45 °C, a melt temperature of 230 °C, and a cooling time of 30 s.

The simulation was performed using Moldflow to analyze the injection, and the velocity and tensor data at each node were extracted. The mesh type was 3D mesh, and the number of elements was 2,616,760. The injection conditions were set to be the same as those of the actual product.

## 4. Results and Discussion

### 4.1. Flake Line of Specimen

**Figure 6:**
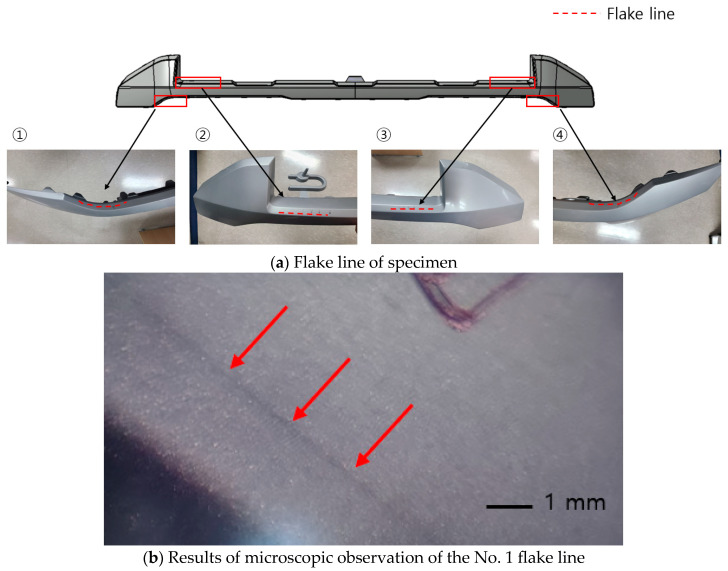
Actual exterior defects. (**a**) The flake line of the specimen, (**b**) the results of the microscopic observation of the No. 1 flake line.

As a result of observing the actual appearance of the product, four defects were identified, two on each side, as shown in Figure 6. When defect No. 1 was observed under a digital microscope (×103), the flake line appeared to be black and in the form of a long line extending from the end of the product.

### 4.2. Velocity Model Result

**Figure 7:**
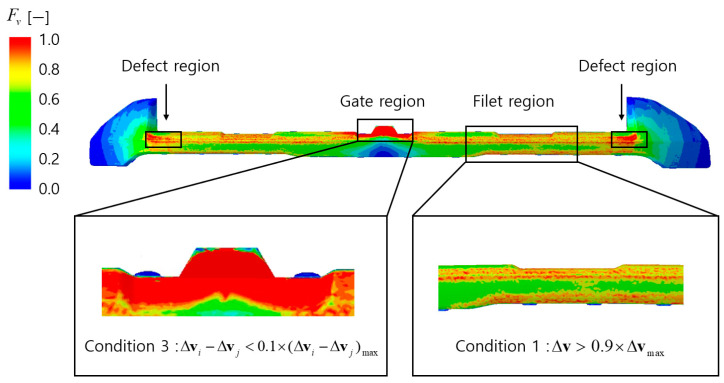
Result of the distribution of the defect condition function $F_{v}$.

The analysis of the $F_{v}$ distribution function, as shown in Figure 7, indicates that high $F_{v}$ values are observed in filet regions, gate regions, and defect regions. In filet regions, abrupt velocity changes caused by the curved geometry result in high $F_{v}$ values. However, as these regions satisfy Condition 1, where the mutual cancelation effect between flow vectors occurs, they are likely to be classified as normal regions.

Therefore, they are expected to be excluded from the final appearance defect prediction results.

The high $F_{v}$ values in gate regions can be attributed to their location experiencing the highest flow velocity throughout the filling process, from the initial to the final stages. However, due to the small average velocity change differences within these regions, resulting in a uniform velocity distribution that satisfies Condition 3, they are classified as defect-free zones. According to Condition 3, regions with minimal average velocity variations, defined as values falling below the lower 10%, are crucial for maintaining a consistent surface appearance by preventing sudden changes in the flake arrangement. Consequently, these regions are excluded from the final appearance defect prediction results.

**Figure 8:**
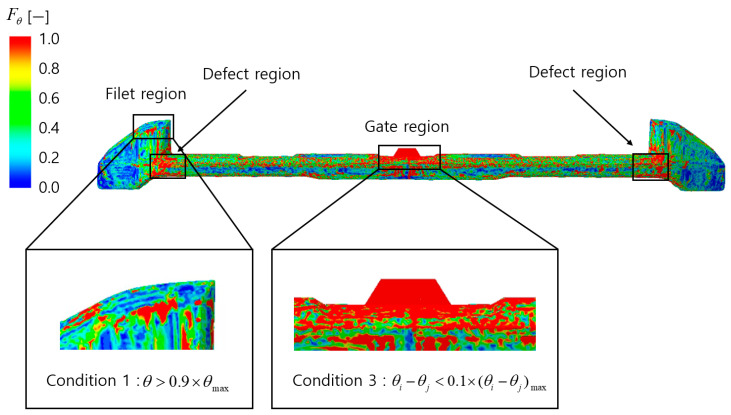
The result of the distribution of the defect condition function $F_{\theta }$.

The analysis of the $F_{\theta }$ distribution function, as shown in Figure 8, reveals high $F_{\theta }$ values in the gate regions, filet regions, and defect regions. In the gate regions, although the $F_{\theta }$ values are high, the small angular difference in average velocity variation satisfies Condition 3, allowing these regions to be classified as normal. In the filet regions, the abrupt velocity changes cause a mutual cancelation effect between flow vectors, satisfying Condition 1, which also allows these regions to be classified as normal.

By applying the normal region conditions, it is expected that only the defect regions will ultimately be observed in the final analysis.

In this study, the analysis of the defect condition function’s distribution in the velocity model was used to evaluate the influence of Δv and θ on appearance defects and to predict normal regions. Since θ is determined by Δv, the defect condition function $F_{v}$ was deemed to have a greater influence on appearance defects than $F_{\theta }$. Accordingly, the defect evaluation function assigned the weights of 1 and 0.5 to α and β, respectively. Conditions for removing normal regions were applied to the defect evaluation function $F_{defect\_velocity}$, and the top 10% of values were classified as defects.

**Figure 9:**
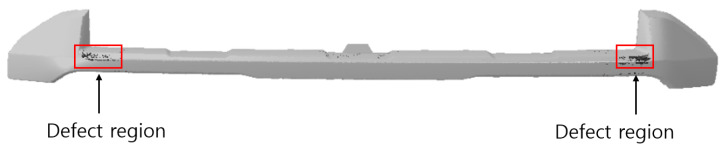
The final appearance defect prediction result of the defect evaluation function $F_{defect\_velocity}$.

Figure 9 presents the final defect prediction results, where actual defects 2 and 3 were observed, achieving an accuracy of 50%. It was confirmed that, as shown in the defect condition function distributions in Figure 7 and Figure 8, normal regions were effectively removed based on the defined conditions, leaving only actual defects. This alignment between the predicted and actual appearance defects demonstrates the validity of the final prediction results and underscores the contribution of this study.

### 4.3. Alignment Model Result

**Figure 10:**
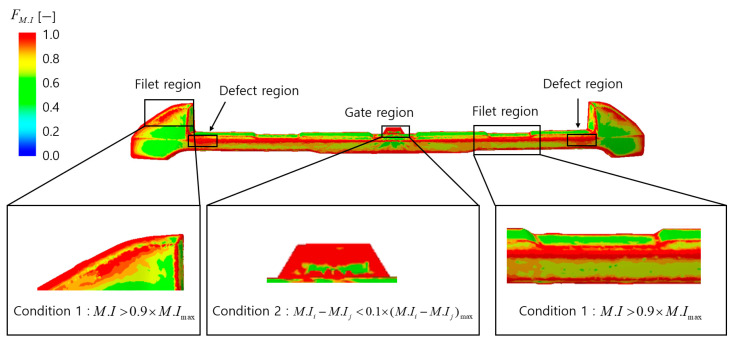
The result of the distribution of the defect condition function $F_{M.I}$.

The analysis of the defect condition function $F_{M.I}$ distribution, as shown in Figure 10, reveals high $F_{M.I}$ values in filet regions, gate regions, and defect regions. In the filet regions, although $F_{M.I}$ values are high, the excessive misalignment caused by curvature and complex flow does not result in significant color differences leading to appearance defects. This satisfies Condition 1, and thus these regions are predicted to be normal.

In the gate regions, there are no abrupt differences in misalignment, which prevents significant color differences and the occurrence of appearance defects. As this satisfies Condition 2, it is predicted that applying the normal region conditions will result in only the defect regions being observed in the final analysis.

**Figure 11:**
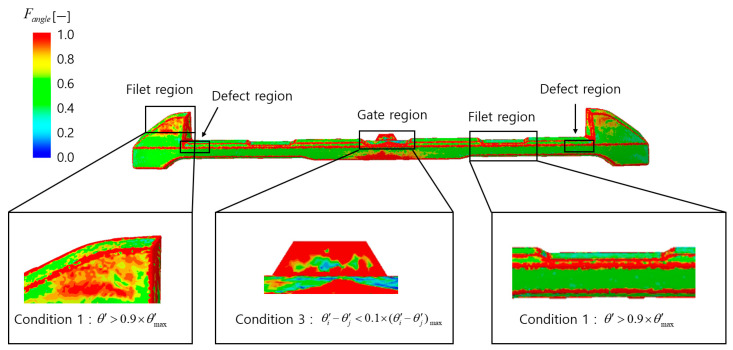
The result of the distribution of the defect condition function $F_{angle}$.

The analysis of the distribution of the defect condition function $F_{angle}$, as shown in Figure 11, indicates high $F_{angle}$ values in the filet regions and gate regions. In the filet regions, although $F_{angle}$ values are high, the excessive angle between the flakes and the surface, caused by curvature and complex flow, makes the occurrence of significant color differences leading to appearance defects unlikely. This satisfies Condition 1, and these regions are predicted to be normal.

In the gate regions, while $F_{angle}$ values are also high, the angle difference between the flakes and the surface within the region does not change abruptly, satisfying Condition 3. As a result, the likelihood of significant color differences is low, and appearance defects are not expected to occur.

In this study, the analysis of the defect condition function distribution in the alignment model was used to evaluate the influence of *M.I*. and $\theta^{\prime }$ on appearance defects and to predict normal regions. Considering that defect-prone regions are not clearly identified in the $F_{angle}$ distribution, *M.I*. was deemed to be a more critical factor for defect prediction than $\theta^{\prime }$. Accordingly, the defect evaluation function assigned weights of 1 and 0.5 to γ and κ, respectively.

**Figure 12:**
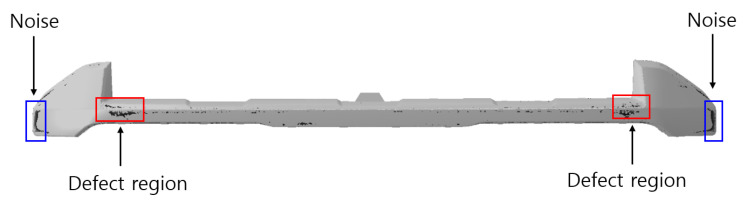
The final appearance defect prediction results of the defect evaluation function $F_{defect\_alignment}$.

The conditions for removing normal regions were applied to the defect evaluation function $F_{defect\_alignment}$, and the top 10% of values were classified as defects. Figure 12 presents the final defect prediction results, where the actual defects 2 and 3 were observed, achieving an accuracy of 50%. It was confirmed that, as shown in the defect condition function distributions in Figure 10 and Figure 11, normal regions were effectively removed based on the defined conditions, leaving only actual defects. This study confirmed that normal regions were effectively excluded as predicted, and the final appearance defect prediction results closely aligned with actual defects, demonstrating the contribution of this research.

However, noise was observed in some areas, particularly near the product edges, where defects do not actually occur. This is likely due to the sensitivity of the condition settings during the defect evaluation process. Future research should focus on reducing noise and improving the precision of the prediction model by refining these conditions.

Despite these limitations, the study successfully predicted actual defect locations, verifying the applicability of the model to real-world products.

## 5. Conclusions

This study presents a novel approach for predicting appearance defects in metallic injection molding processes, demonstrating its applicability in industries requiring high-quality metallic finishes, such as automotive interiors and consumer electronics. The proposed model can enhance manufacturing efficiency, reduce defect rates, and contribute to cost reduction and quality improvement. Given the importance of consistent metallic luster and surface quality in the automotive and consumer electronics industries, the findings of this research significantly increase the potential for industrial application.

In this study, two flake line defect prediction models were proposed, considering the flow velocity differences between surface and core layers, aluminum flake alignment, and the angle between flakes and the surface. These models quantitatively analyze the influence of surface-core interactions on appearance defects, enabling a clearer understanding of defect formation mechanisms and the development of more refined predictions and preventive measures.

Unlike previous studies, which focused on defect prediction in simplified specimens, this research developed a methodology applicable to actual products, marking a significant advancement. Notably, the proposed approach successfully predicted two out of four defects with an accuracy of 50%, demonstrating the reliability of the models through consistent predictions across both models.

However, challenges remain, particularly the issue of noise when using the Alignment model. Addressing this requires advancements in filtering techniques during data pre-processing, as well as improving and adding filtering conditions to enhance prediction accuracy. Additionally, optimizing coefficient settings is essential to further improve the accuracy and reliability of the models. Verifying the generalizability of the methodology across various geometries and material conditions is another critical area for future research.

To achieve this, further experimental validation and simulations across diverse industrial applications are needed. Moreover, integrating data-driven machine learning techniques offers promising potential in terms of enhancing predictive performance. Machine learning can leverage large datasets to identify patterns, thereby improving the accuracy and reliability of defect prediction.

## Data Availability

The original contributions presented in this study are included in the article. Further inquiries can be directed to the corresponding author.
